# A Novel Multi-Approach Protocol for the Characterization of Occupational Exposure to Organic Dust—Swine Production Case Study

**DOI:** 10.3390/toxics6010005

**Published:** 2017-12-27

**Authors:** Carla Viegas, Tiago Faria, Ana Monteiro, Liliana Aranha Caetano, Elisabete Carolino, Anita Quintal Gomes, Susana Viegas

**Affiliations:** 1 GIAS, ESTeSL—Escola Superior de Tecnologia da Saúde de Lisboa, Instituto Politécnico de Lisboa, 1990-096 Lisbon, Portugal; tiagofaria@ctn.tecnico.ulisboa.pt (T.F.); ana.monteiro@estesl.ipl.pt (A.M.); Liliana.caetano@estesl.ipl.pt (L.A.C.); etcarolino@estesl.ipl.pt (E.C.); anita.gomes@estesl.ipl.pt (A.Q.G.); susana.viegas@estesl.ipl.pt (S.V.); 2 Centro de Investigação em Saúde Pública, Escola Nacional de Saúde Pública, Universidade NOVA de Lisboa, 1600-560 Lisbon, Portugal; 3 Centro de Ciências e Tecnologias Nucleares, Instituto Superior Técnico, Universidade de Lisboa, E.N. 10 ao km 139,7, 2695-066 Bobadela LRS, Portugal; 4 Research Institute for Medicines (iMed.ULisboa), Faculty of Pharmacy, University of Lisbon, 1649-004 Lisbon, Portugal; 5 Faculty of Medicine, University of Lisbon Institute of Molecular Medicine, 1649-028 Lisbon, Portugal

**Keywords:** organic dust, occupational exposure, swine, culture-based methods, molecular tools

## Abstract

Swine production has been associated with health risks and workers’ symptoms. In Portugal, as in other countries, large-scale swine production involves several activities in the swine environment that require direct intervention, increasing workers’ exposure to organic dust. This study describes an updated protocol for the assessment of occupational exposure to organic dust, to unveil an accurate scenario regarding occupational and environmental risks for workers’ health. The particle size distribution was characterized regarding mass concentration in five different size ranges (PM0.5, PM1, PM2.5, PM5, PM10). Bioburden was assessed, by both active and passive sampling methods, in air, on surfaces, floor covering and feed samples, and analyzed through culture based-methods and qPCR. Smaller size range particles exhibited the highest counts, with indoor particles showing higher particle counts and mass concentration than outdoor particles. The limit values suggested for total bacteria load were surpassed in 35.7% (10 out of 28) of samples and for fungi in 65.5% (19 out of 29) of samples. Among *Aspergillus* genera, section *Circumdati* was the most prevalent (55%) on malt extract agar (MEA) and *Versicolores* the most identified (50%) on dichloran glycerol (DG18). The results document a wide characterization of occupational exposure to organic dust on swine farms, being useful for policies and stakeholders to act to improve workers’ safety. The methods of sampling and analysis employed were the most suitable considering the purpose of the study and should be adopted as a protocol to be followed in future exposure assessments in this occupational environment.

## 1. Introduction

During the past few years, most animal husbandry practices in Europe and the United States have been industrialized, resulting in livestock operations raising thousands of animals in a single facility [1,2]. The confined production of swine promotes waste by-products and feed concentration, with the subsequent generation of high levels of organic dust, including dusts, gases, microorganisms, microbial metabolites and other potential airborne health hazards [1].

The bioburden, comprising fungi and bacteria, should be considered among organic dust as an important airborne pollutant in swine production facilities [3]. Organic dust in swine production may be generated by various microbial growth substrates, such as feeding materials [4,5], moldy hay, feces [6,7,8,9], manure and bedding material [10], ventilation, filtration and misting applications [11]. This diversity of contamination sources worsens the occupational problem and hinders the effectiveness of control measures [8]. Of note, this occupational environment may be associated with high aerosolization of particulate matter, boosting exposure to microorganisms such as fungi [8,9] and to the mycotoxins produced by them [8,10].

Swine workers have an increased prevalence of several respiratory symptoms and diseases, such as chronic bronchitis, chronic obstructive pulmonary disease and organic dust toxic syndrome [9,12,13,14,15,16]. In addition, work practices such as the types and methods of swine feeding, the use of wood shavings for animal bedding and the use of some specific disinfectants have also been related to workers’ symptoms [8,10,17,18,19]. When feeding occurs, abundant organic dust particles from feed become aerosolized for a long time or deposited on the floor [10]. High dispersion of microorganisms and their metabolites also occurs as they are resuspended during the feeding task [20,21]. 

Although the need for manual work in swine production is decreasing, mostly due to the confinement of swine production to facilities with automation, in Portugal, as in other countries, some activities still require farmers’ direct intervention, such as piglet tail cut and vaccination, among other activities related to swine breeding [8]. In these activities, increased occupational exposure to organic dust occurs [5,8,9], as previously reported for fungal contamination [5], mycotoxins [8] and particulate matter [9] in Portuguese swine farms. In this study, a different protocol for the assessment of exposure to organic dust is described, implemented to unveil a more accurate occupational exposure scenario regarding the organic dust risk factor. In fact, besides the assessment of particles, bacteria and fungi in air and surface samples, molecular tools were extensively applied to target harmful fungal species, and feed samples were also screened.

## 2. Materials and Methods

### 2.1. Swine Farms’ Characteristics and Collection of Environmental Samples

Five Portuguese swine farms were prospected in the Lisbon district between June and July of 2017. Swine farms were selected according to the following criteria: location within Lisbon district, highest number of animals (Table 1) and number of workers. The five farms had been evaluated, among others, in an earlier study from our group [5], with specific recommendations on the need to implement safety measures in the context of environmental and occupational health. However, no modifications in working activities or safety procedures were observed. 

In the swine farm A, maternity was the main activity, despite having other areas with on-going activities. The floor in the swine maternities was covered with journal paper. Manure removal systems were present in all farm facilities, with complete removal from the building several times a day. The ventilation systems in the studied farm buildings consisted of mechanical ventilation by wall exhaust fans coupled with natural ventilation through the operation of a winch-curtain. Farm workers did not use respiratory protection devices in any of the farms.

Twenty to twenty-five air samples from five distinct areas (pig gestation site, maternity, stalls, pig fattening area and quarantine confinement) (Table 1) and one outdoor air sample (to be used as a reference) were taken in each swine farm. Samples from surfaces (wall swab), floor coverage from maternities (journal paper) and feed from pig gestation site and/or pig fattening were also collected at each farm (Table 1).

### 2.2. Particulate Matter Assessment

Particle measurement was performed with direct-reading equipment (Handheld Particle Counter from Lighthouse Worldwide Solutions (Model 3016/5016)). This measurement equipment gives information regarding particle mass concentration (mg × m^−3^) (PMC) at five different sizes (PM0.5, PM1, PM2.5, PM5, PM10). Particle counts (PNC) by particle diameter size were also obtained with the same equipment for six different diameters (0.3 μm, 0.5 μm, 1 μm, 2.5 μm, 5 μm and 10 μm). Measurements were performed near the nose of each worker under typical ventilation conditions and during the development of tasks performed in each of the five areas of interest.

### 2.3. Bioburden Sampling and Analysis by Culture-Based Methods

Air samples (50 L) were collected at a 1-m height onto four media: 2% malt extract agar (MEA) with 0.05 g/L chloramphenicol media; dichloran glycerol (DG18) agar-based media; tryptic soy agar (TSA) with 0.2% nystatin; Violet Red bile agar (VRBA), with a flow rate of 140 L of air per minute. For surface samples, the walls of the considered indoor locations were swabbed using a 10 by 10 cm square stencil, disinfected with 70% alcohol solution between samples, according to the International Standard ISO 18593-2004, and swabs were plated onto the selected media. 

For floor coverage and feed samples, 4.4 g of each (not oven-dried prior to processing, thus retaining natural water content) were washed in 40 mL of sterilized distilled water for 20 min at 200 rpm, and 0.15 mL of this suspension were spread onto the four media. After incubation of MEA and DG18 at 27 °C for 5 to 7 days for fungi and TSA and VRBA at 30 °C and 35 °C for 7 days for mesophilic bacteria and coliforms (Gram-negative bacteria), respectively, bioburden densities (colony-forming units, CFU·m^−3^, CFU·m^−2^, CFU·g^−1^) were calculated. Fungal species were identified microscopically using tease mount or Scotch tape mount and lactophenol cotton blue mount procedures. Morphological identification was achieved through macro- and microscopic characteristics [22]. 

### 2.4. Fungal Sampling and Molecular Detection by Real-Time PCR

Air samples (300 L) were collected using the impinger Coriolis μ air sampler (Bertin Technologies) with a flow rate of 300 L of air per minute. Samples were collected onto 10-mL sterile phosphate-buffered saline (PBS) with 0.05% Triton X-100, and an aliquot was subsequently used for DNA extraction using the ZR Fungal/Bacterial DNA MiniPrep Kit (Zymo Research, Irvine, CA, USA) according to the manufacturer’s instructions. Aliquots of surface samples, floor coverage (journal paper) and feed samples were also used for DNA extraction following the same steps as the impinger samples (Table 1).

A Viia7 qPCR Detection System (Thermo Fisher Scientific, Waltham, MA, USA) was used to perform real-time PCR (RT-PCR) for the molecular detection of *Aspergillus* species/strains (Table 2). Reactions included 1× iQ Supermix (Bio-Rad, Amadora, Portugal), 0.5 μM of each primer and 0.375 μM of TaqMan probe in a total volume of 20 μL. Amplification followed a three-step PCR: 40 cycles with denaturation at 95 °C for 30 s, annealing at 52 °C for 30 s and extension at 72 °C for 30 s. A non-template control was used in every PCR reaction. As positive controls for the species, DNA samples were obtained from reference strains from the Mycology Laboratory from the National Institute of Health Doutor Ricardo Jorge (INSA).

### 2.5. Statistical Analysis

The software SPSS, Version 24.0 for Windows, IBM, Lisbon, Portugal, 2016 was used for statistical analysis. The results were considered significant at the 5% significance level. The Shapiro–Wilk test was used to test data normality. Frequency analysis (*n*, %) was used for the qualitative data. Minimum, maximum, median and interquartile range were determined in the quantitative data, since normality was not verified. The Friedman test was used to compare the particle concentration of different dimensions (either the counts or the mass), since the normality assumption was not verified. The Kruskal–Wallis test was used to compare the concentration of particles of different sizes (either counts or mass) between the five swine farms studied and between workplaces, since the normality assumption was not verified. The Kruskal–Wallis test was used to compare fungi and bacteria concentration, both in air and on surfaces, between the five swine farms studied and between workplaces, since the normality assumption was not verified. Spearman’s correlation coefficient was used to study the relationship between fungi and bacteria concentration (air and surface) and particle concentration (counts and mass).

## 3. Results

### 3.1. Particulate Matter

Statistically-significant differences were detected for particle counts (PNC) of different dimensions and for particle mass concentrations (PMC) of various dimensions ($\chi_{F}^{2}$(4) = 120.000, *p* = 0.000) (Friedman’s multiple comparisons). Smaller particles (0.3 µm) were the ones with significantly higher counts and lower mass concentrations.

Smaller particle (0.3 µm) counts were significantly different among swine farms ($\chi_{K-W}^{2}$(4) = 27.832, *p* = 0.000), particularly among Swine Farm D and Swine Farms A (*p* = 0.002) and E (*p* = 0.000), and among Swine Farms C and E (*p* = 0.005). Regarding the counts of 0.5 µm-sized particles, significant differences were also found between swine farms ($\chi_{K-W}^{2}$(4) = 25.353, *p* = 0.000), namely among Swine Farm D and Swine Farms A (*p* = 0.000) and E (*p* = 0.000). Through mean ranks analysis, it was found that 0.3 µm- and 0.5 µm-sized particle counts were significantly higher in Swine Farm E, followed by Swine Farm A. No statistically-significant differences (*p* > 0.05) were detected for particle counts of the remaining sizes.

Regarding particle mass results, the PM0.5 concentration was significantly different between swine farms ($\chi_{K-W}^{2}$(4) = 27.832, *p* = 0.000), namely among Swine Farms D and Swine Farms A (*p* = 0.002) and E (*p* = 0.000) and among Swine Farms C and E (*p* = 0.005). Higher mass concentrations of PM0.5 particles were observed in Swine Farms E and A. The differences found for PM1.0 concentrations between swine farms ($\chi_{K-W}^{2}$(4) = 27.389, *p* = 0.000) followed the same trend, i.e., Swine Farm D differed significantly from Swine Farms A (*p* = 0.001) and E (*p* = 0.000) and C differed from E (*p* = 0.008). Swine Farms E and A also exhibited the highest mass concentrations of PM1.0 particles. As for the mass concentration of the particles PM2.5, statistically-significant differences were also detected among the swine farms ($\chi_{K-W}^{2}$(4) = 13.159, *p* = 0.011), namely among Swine Farms D and A (*p* = 0.007), with the highest concentrations observed in Swine Farm A. No statistically-significant differences (*p* > 0.05) were detected for mass concentration of the remaining particles. 

Higher values of particle counts and particle mass concentration were found indoor, in comparison to outdoor air samples. No statistically-significant differences were found among the work areas (maternity, gestation, batteries, fattening and quarantine), neither in counts nor on mass concentrations of the different particle sizes (*p* > 0.05). 

### 3.2. Bioburden: Bacterial Contamination

**Figure 1:**
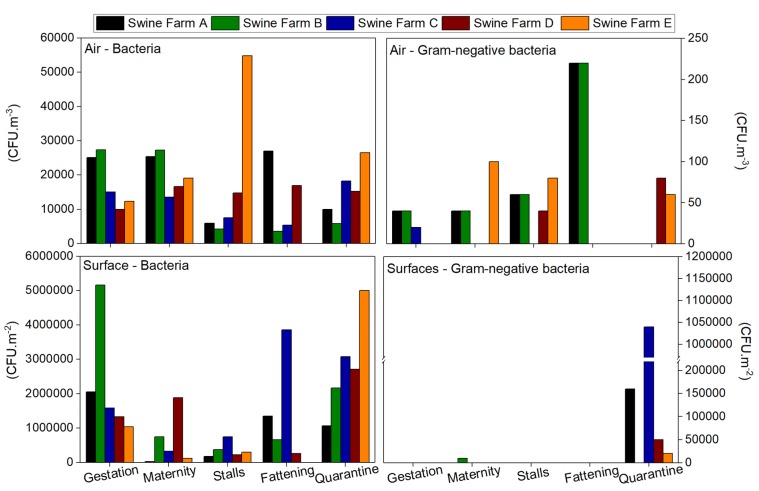
Bacterial load obtained for air and surface samples.

Results for total bacterial load in indoor air ranged from 1800 to 54,840 CFU·m^−3^, with Swine Farm E presenting the highest median value (28,210 CFU·m^−3^) Swine Farms A, B, C and D with mean values of 18,688, 13,660, 11,944 and 14,720 CFU·m^−3^, respectively. In 35.7% (10 out of 28) of the sampling sites, the total bacterial load exceeded the limit values already suggested [25] (10,000 CFU·m^−3^ for total bacteria for eight hours of work for agricultural environments). Gram-negative bacteria load in the air ranged between 0 and 220 CFU·m^−3^ with the highest median value (72 CFU·m^−3^) found in Swine Farms A and B, followed by Swine Farms C, D and E, with mean values of 4, 24 and 60 CFU·m^−3^, respectively. None of the sampled sites exceeded the limit values suggested by Goyer [25] (1000 CFU·m^−3^ for Gram-negative bacteria for eight hours of work for agricultural environments) (Figure 1). The bacterial load in outdoor air was lower than in indoor air in each swine farm, except for the “fattening” area in Farm C, presenting a slightly lower value indoors than the total bacterial load outdoors.

Regarding surface samples, mesophilic bacterial population ranged from 3 × 10^4^ to 516 × 10^4^ CFU·m^−2^, with the highest median value found in Swine Farm C (192 × 10^4^ CFU·m^−2^), followed by Swine Farms A, B, D and E with mean values of 93.4 × 10^4^, 182.4 × 10^4^, 128.4 × 10^4^ and 162 × 10^4^ CFU·m^−2^, respectively. Gram-negative bacteria ranged between 0 and 104 × 10^4^ CFU·m^−2^, with only one sampling site in each swine farm presenting coliform growth (Gram-negative bacteria), mainly in the “quarantine” and in the “maternity” area in Swine Farm B. 

Feed bacterial contamination ranged between 81 and 1237 CFU·g^−1^, and the “maternity” from Swine Farm E showed countless mesophilic bacteria. Gram-negative bacteria concentrations ranged from 0 to 363 CFU·g^−1^, and the same sampling site showed countless colonies, whereas in the two other sampling sites, isolates were not observed (“stalls” in Swine Farm A and “quarantine” in Swine Farm C). 

Concerning bedding results, countless mesophilic bacteria were present in all swine farms, except in Farm D with a value of 1529 CFU·g^−1^. Regarding Gram-negative bacteria, swine Farm E presented countless coliforms, and the other swine farms ranged between 489 and 1552 CFU·g^−1^. 

These results suggest a higher contribution of Gram-positive than Gram-negative bacteria in the bacteriota load.

### 3.3. Bioburden: Fungal Contamination

Fungal load in indoor air ranged from 40 to 3120 CFU·m^−3^ on MEA, with Swine Farm E presenting the highest median value (2500 CFU·m^−3^), followed by Swine Farms A, B, C and D with mean values of 124, 140, 604 and 104 CFU·m^−3^, respectively. Noteworthy, 65.5% (19 out of 29) of the sampling sites showed higher fungal load than the limits imposed by the World Health Organization (WHO) (maximum value of 150 CFU·m^−3^) (2). Two out of the 29 (6.9%) air samples collected in the five swine farms presented higher fungal load when compared to the outdoor sampling. Swine Farm A was the exception, with higher fungal load indoors (two out of five collected samples).

**Figure 2:**
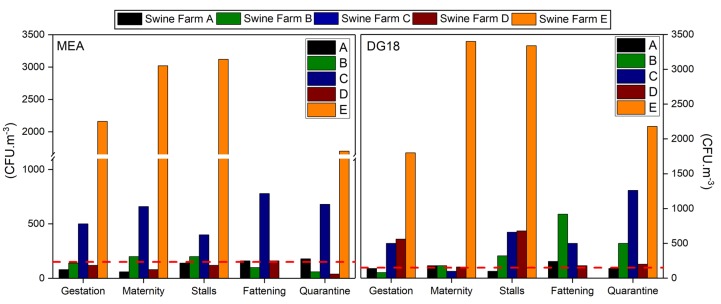
Fungal load distribution in the five assessed swine farms. The dashed line represents the reference limits suggested by the World Health Organization (WHO).

Similar results were found on DG18, with fungal load ranging from 80 to 3400 CFU·m^−3^ and with Swine Farm E also presenting the highest median value (2680 CFU·m^−3^), followed by Swine Farms A, B, C and D, with mean values of 160, 400, 604 and 356 CFU·m^−3^, respectively. DG18 revealed an increased amount of sampling sites (82.8%; 24 out of 29) with fungal load exceeding the WHO limits (maximum value of 150 CFU·m^−3^) (Figure 2). Nine out of the 29 (31%) air samples presented higher indoor fungal load when compared to the outdoor sampling. Swine Farm B was the one with the highest number of air samples presenting higher load indoors (four out of five collected samples).

Twenty five different fungal species were found in air samples on MEA and 18 on DG18. *Cladosporium* sp. was the most prevalent in indoor air samples in both media (59.4% MEA; 66.5% DG18), followed by *Fusarium graminearum* species complex (13.2%) on MEA and *Ulocladium* sp. (14.6%) on DG18 (Table 3). 

Fungal load in substrate (feed and floor coverage) and surface samples was distributed as follows: 0 to 4 CFU·g^−1^ (MEA) and 0 to 39 CFU·g^−1^ (DG18) in feed; 0 to 1 CFU·g^−1^ (MEA) and no isolates (DG18) in floor covering; 0 to 59 × 10^4^ CFU·m^−2^ (MEA) (maximum values of 14 × 10^4^ CFU·m^−2^ in Swine Farm D, followed by 6 × 10^4^ CFU·m^−2^ in Swine Farm E) and 0 to 370,500 (DG18) (maximum values of 10 × 10^4^ CFU·m^−2^ in Swine Farm D) on wall surfaces.

Three different fungal species were found in feed samples on both media, the *Cladosporium* genus being the most prevalent (71.4% MEA; 82.2% DG18). Fungi isolates from floor covering samples were only observed on MEA, with predominant *Penicillium* genus (50.0%) followed by *Alternaria* sp. (37.5%). Ten fungal species were found in surface samples on MEA and four on DG18. *Cladosporium* sp. (53.8%) and *Scopulariopsis brevicaulis* (33.3%) were the most detected species on MEA, whereas *Scopulariopsis candida* (50.3%) and *Aspergillus* section *Circumdati* (19.9%) were the most observed on DG18. *Trichothecium roseum* was found only on surfaces (Table 3).

*Aspergillus* genera were observed on MEA and DG18 (2.26%). *Aspergillus* section *Circumdati* was the most prevalent (55%) on MEA followed by *Aspergilli* (25%). Different *Aspergillus* sections were more prevalent on DG18, *Versicolores* being the most identified (50%) followed by *Usti* (20.8%) (Table 4).

No *Aspergillus* section *Fumigati* nor *Aspergillus* section *Versicolores* were detected by qPCR.

### 3.4. Correlation and Comparison Results

No statistically-significant differences were detected between the swine farms in either surface or air samples, regarding total bacterial load ($\chi_{K-W}^{2}$(4) = 1.936, *p* = 0.748, $\chi_{K-W}^{2}$(4) = 3.676, *p* = 0.452, respectively) or Gram-negative bacteria ($\chi_{K-W}^{2}$(4) = 0.081, *p* = 0.999, $\chi_{K-W}^{2}$(4) = 7.132, *p* = 0.129, respectively).

Statistically-significant differences were found between the fungal load on MEA in the swine farms in surface ($\chi_{K-W}^{2}$(4) = 13.699, *p* = 0.008) and in air ($\chi_{K-W}^{2}$(4) = 14.602, *p* = 0.001) samples. Fungal load was significantly different among Swine Farms C and D (*p* = 0.036) in surface samples and among Swine Farm D and Swine Farms A (*p* = 0.007) and B (*p* = 0.025) in air samples. Swine Farm D presented the highest mean rank values for fungal load in both surface and air samples. Statistically-significant differences were also observed for fungal load on DG18 between the air samples collected in the swine farms ($\chi_{K-W}^{2}$(4) = 12.621, *p* = 0.013), namely, between Swine Farms B and D (*p* = 0.005), with Farms D and B exhibiting the highest and the lowest values, respectively (Table 5).

Among the sampling sites (maternity, gestation, battery, fattening and quarantine), no statistically-significant differences were detected in fungi nor in bacteria load of either surface or air samples (*p*’s > 0.05).

The relationship between fungi, bacteria (surface and air) and particles’ concentration (counting and mass) was performed separately for each swine farm. In Swine Farm A, a significant negative correlation, with strong intensity, was found between the fungal concentration on DG18 in surface samples and the fungal concentration on MEA in the air (r_s_ = −0.975, *p* = 0.005). This result indicates that higher fungal concentrations on DG18 in the surface are related to lower fungal concentrations on MEA in the air. In Swine Farm B, higher total bacteria loads in surface samples were found to be significantly correlated with lower counts of 0.5 micron (r_s_ = −0.9, *p* = 0.037), 1.0 micron (r_s_ = −0.9, *p* = 0.037), 2.5 micron (r_s_ = −0.9, *p* = 0.037), 5.0 micron (r_s_ = −0.9, *p* = 0.037) and 10.0 micron (r_s_ = −0.9, *p* = 0.037) particles. Additionally, higher fungal loads on DG18 in surface samples were found to be positively correlated with higher fungal loads on MEA in surface samples (r_s_ = 0.918, *p* = 0.028) and with higher total bacteria loads in the air (r_s_ = 0.894, *p* = 0.041). A statistically-significant correlation was also found in Swine Farm B between higher PM0.5 mass concentration and higher fungal loads on MEA in the air (r_s_ = 0.9, *p* = 0.037). In Swine Farm C, total bacteria load in the air was found to be negatively correlated with fungal load on DG18 in the air (r_s_ = −0.9, *p* = 0.037), suggesting that higher concentrations of total bacteria in the air are related to lower concentrations of fungi in the air. No significant correlations were found for Swine Farms D and E. 

## 4. Discussion

Organic dust has been the focus of several epidemiological studies, as exposure to organic dust is described as causing mucous membrane irritation in the eyes and upper and lower airways, inflammation by allergic and non-allergic mechanisms and airway obstruction [26]. Organic dust in swine production can be generated from several sources such as feed, skin cells, hair and dried feces. Acute exposures to high levels of dust may provoke increased phlegm production and pulmonary inflammation 4−10 h after exposure. In addition, chronic exposures may result in bronchitis and asthma [27]. 

It has already been reported that aerosols produced in swine production facilities may have particle size distributions considerably smaller than 1.0 µm in diameter [28,29], comprising an important size fraction of the respirable aerosols that should be assessed in swine production [28,29]. Furthermore, particulate matter can be the vehicle for the bioburden present in swine farms, reaching workers respiratory systems and, consequently, enhancing the occupational exposure of swine workers to organic dust [9]. Therefore, microorganisms adhering to particulate matter, along with coexisting gases and toxins are a general cause of concern regarding co-exposure to several risk factors and possible additive and synergistic health effects [20,30]. This scenario reinforces the theory that occupational exposure is rarely associated with a single factor, since it is commonly a combination of several risk factors [31].

It has been suggested that stationary samplers can be adequately used in the assessment of personal exposure to airborne fungi in confined agricultural environments [7] as is the case of animal production. However, we should combine not only more than one active method for air sampling [32,33], but also couple it with passive methods [34,35], such as surface swabs, floor covering and feed to obtain a more accurate risk characterization [33]. As such, this study was performed using a multi-approach protocol for the characterization of occupational exposure to organic dust by applying the impaction method to obtain a bioburden viable fraction from organic dust, the impinger method for the detection of harmful fungal species and the surface swabbing to complement air samples’ results regarding the diversity of the bioburden [31,35]. In addition, the collection of floor covering and litter (besides surfaces swabs) samples allowed determining the contamination levels from a larger period of time (weeks to several months), whereas air samples can only reflect the load from a shorter period of time (mostly minutes) [33,34,36].

Regarding particles’ results, the differences obtained between indoors and outdoors demonstrate that emission sources are located indoors and are directly related to the tasks developed by swine workers. The fact that smaller particles (0.3 µm) had higher counts indoors suggests that exposure of workers to particles can potentially induce health effects in the upper and lower respiratory systems, as smaller particles can reach the pulmonary alveoli. This trend of results was previously reported by Viegas et al. (2016), describing the presence of higher values of the smaller particles in several occupational settings (cork industry, bakeries, horse stables and waste management) [37]. Particles are also recognized to be an important carrier of other pollutants that cannot reach the respiratory system per se, as is the case of mycotoxins (since these molecules are not volatile), already reported in previous studies where occupational exposure to aflatoxin B1 was observed [8]. Moreover, several fungal species with recognized toxigenic potential [38] were found in the different environmental matrices considered in this study. Of note, different *Aspergillus* sections, *Fusarium graminearum* species complex, *Trichothecium roseum*, among others, were identified. 

Depending on the species, fungal spores can remain aerosolized during a long time due to fungal ability to produce dry spores [39]. This is the case of *Cladosporium* sp., the most prevalent fungi found in the assessed swine farms. The *Aspergillus* genera also belong to the dry spore-producing fungi group, with *Aspergillus* species having dry, light spores that are resistant to desiccation, which greatly enhance their ability to disseminate in indoor occupational environments [40,41]. This aspect can explain the relation obtained between fungal load with higher PM0.5 particle concentration (Swine Farm B) and the fact that Swine Farm E presented the highest particle counts and also the highest fungal load. Another factor that should be considered is animal density, reported as one of the environmental variables that greatly influences the generation of particulate matter and bioburden in swine production facilities [42,43]. Other variables that can boost exposure to organic dust in swine production are the stage of the animals’ growth, the manure management procedures and the floor coverage used, among others [42,43].

These last aspects can explain the fact that Swine Farm A obtained higher exposure values for both exposure metrics used to assess particles contamination, since it was the only assessed farm with maternity as the principal activity. In maternity-type facilities, important and specific features are present, such as a high percentage of floor coverage with paper and other soft materials in the production areas in order to promote a more pleasant environment for the small animals. Furthermore, specific feed and different tasks are developed to guarantee more specific sanitary conditions and to take care of the small animals, such as cutting piglets’ tails and vaccination, which increase the time spent by workers in the pig confinement facilities [5]. These specifications can enhance particle emission and resuspension, in association with bioburden dispersion, as explained by some of the correlations found and, consequently, increase occupational exposure to organic dust [9]. 

Concerning bacteria load, the results obtained from the different environmental matrices indicate that more than one third of the sampling sites presented higher total bacteria load than the suggested limit values. Furthermore, the indoor air presented a higher bacterial load than outdoors, proving the indoor contamination sources [5,44]. Moreover, the feed and bedding bacteria contamination can constitute indoor contamination sources boosting bacteria dissemination indoors [4,5]. Finally, due to the Gram-negative bacteria presence in all the analyzed matrices, occupational exposure to endotoxins should be considered [45]. Of note, the increased bacteriota load compared with fungal load is in line with other studies within the same occupational environment [1,6,46,47]. Furthermore, in another study, Gram-positive bacteria dominance in the microbial flora from Swedish swine confinement buildings was reported. In this case, higher *Enterococci* prevalence should be also expected [1].

When comparing the results of the fungal load through MEA and DG18 assessment, DG18 allowed the identification of a higher number of samples surpassing WHO limit values with higher indoor fungal load when compared with the outdoor load, whereas with MEA, a higher diversity of the mycobiota was observed, and it was possible to recover contamination from the floor covering. The complementarity of the results allows us to infer the importance of using both media to achieve a more accurate mycobiota exposure assessment, and this should be considered when planning an assessment in this specific occupational environment. As in other studies [5,48] developed in settings with high fungal contamination, it was possible to identify one fungal species on surfaces that was not found in air samples (*Trichothecium roseum*), justifying the inclusion of this passive method in assessment protocols intended to ensure accurate assessment of exposure to mycobiota [33].

*Scopulariopsis* species were among the most prevalent fungal species found on the surfaces in the assessed swine farms, corroborating the results obtained in a previous study [5]. *Scopulariopsis brevicaulis* has been related to cases of occupational exposure to fungi, and several species from this genus are also known to produce opportunistic infections such as onychomycosis. Therefore, surface contamination potentially represents an increased risk factor for workers’ health [45], since this fungal species is a common saprophytic fungi in pigs [49].

The *Aspergillus* sections analyzed by molecular tools were selected due to previous studies developed in Portuguese swine production reporting *Aspergillus* section *Versicolores* as the most prevalent section from the genus [5,50]. Considering *Aspergillus* section *Fumigati,* besides its clinical relevance [51,52], this species was observed to be predominant and detected in several sampling sites, only by molecular tools, in other critical Portuguese occupational environments [32,33,34,53,54]. Furthermore, both *Aspergillus* sections detected are representative of harmful fungal species, and their presence requires the implementation of corrective measures according to the American Industrial Hygiene Association [55].

*Aspergillus* section *Fumigati* was not detected by conventional methods, nor by qPCR, strongly indicating that this species complex is not present in the samples analyzed. The same is not true for *Aspergillus* section *Versicolores* that was detected by conventional tools as in the previous study [5] and not by qPCR. The lack of qPCR detection in this case could be due either to ineffective release of microbial DNA content from cells, or poor DNA recovery after extraction and purification steps [56], or to the presence of inhibitors, namely the presence of particles in the air as previously reported [57]. The same drawbacks can be present even with more refined analytical tools, based on next-generation sequencing (NGS) technologies [54]. Furthermore, the NGS is a qualitative method, and to get a better understanding of the bioburden exposure, the concentration must be assessed using culture-based methods and molecular biology methods such as qPCR [54]. This approach will allow: by using culture-based methods to obtain information about the infection potential of the bioburden present [58] and comparing quantitative information with guidelines; by applying molecular tools to target specific species indicators of harmful bioburden and to overcome some culture-based methods constraints [30]. 

## 5. Conclusions

Swine farms are prone to the presence of several occupational risk factors. Workers in these settings have a cumulative exposure to, at least, fungi, bacteria, their metabolites and particles, all of these comprising organic dust. This multiple, diverse and constantly changing contamination has to be considered in the risk assessment process, and preventive and protective measures should be able to provide an answer to all these exposure characteristics. The sampling (active and passive) and analysis (culture-based and molecular) methods employed were the most suitable considering the study goal and should be adopted as a protocol to be followed in future exposure assessments in this occupational environment. This study unveiled a wider occupational exposure scenario characterization regarding organic dust, and it will be useful for policies and stakeholders to act to improve workers’ safety conditions. 

## Figures and Tables

**Table 1:** Number of samples collected and animal quantity in each farm.

Swine Farms	No. of Air Samples Impaction *	No. of Air Samples Impinger	No. of Surfaces Samples (Walls)	No. of Feed Samples	No. of Floor Cover Samples	Animal Quantity
A	20	5	5	2	1	1768
B	20 #	5	5	2	1	8000
C	20	4 #	5	2	1	3300
D	20	5	5	2	1	6000
E	16 +	4	4	2	1	7000

* At each working site, 4 air samples were taken for each media (malt extract agar (MEA), dichloran glycerol (DG18), tryptic soy agar (TSA), Violet Red bile agar (VRBA)); +: farm without pig fattening; #: one sample lost.

**Table 2:** Sequence of primers and TaqMan probes used for real-time PCR.

*Aspergillus* Sections Targeted	Sequences	Reference
*Fumigati*		
Forward Primer	5′-CGCGTCCGGTCCTCG-3′	
Reverse Primer	5′-TTAGAAAAATAAAGTTGGGTGTCGG-3′	Cruz-Perez et al. 2001 [23]
Probe	5′-TGTCACCTGCTCTGTAGGCCCG-3′	
*Versicolores*		
Forward Primer	5′-CGGCGGGGAGCCCT-3′	
Reverse Primer	5′-CCATTGTTGAAAGTTTTGACTGATcTTA-3′	
Probe	5′-AGACTGCATCACTCTCAGGCATGAAGTTCAG-3′	EPA 2017 [24]

**Table 3:** Fungal distribution in environmental and substrate matrices after inoculation onto MEA and DG18 media.

MEA		DG18	
**Air**	**(CFU·m^−3^) (%; *n*)**	**Air**	**(CFU·m^−3^) (%; *n*)**
*Cladosporium* sp.	59.4; 12,100	*Cladosporium* sp.	66.5; 14,120
*Fusarium graminearum*	13.2; 2700	*Ulocladium* sp.	14.6; 3100
*Alternaria* sp.	5.7; 1160	*Chrysonilia sitophila*	4.7; 1000
Others	21.7; 4420	Others	14.2; 3020
**Surfaces**	**(CFU·m^−2^) (%; *n*)**	**Surfaces**	**(CFU·m^−2^) (%; *n*)**
*Cladosporium* sp.	53.8; 210,000	*Scopulariopsis candida*	50.3; 580,000
*Scopulariopsis brevicaulis*	33.3; 130,000	*Aspergillus* section *Circumdati*	19.9; 230,000
*Penicillium* sp.	12.8; 50,000	*Cladosporium* sp.	13; 150,000
Others	0.1; 500	Others	16.7; 193,000
**Feed**	**(CFU·g^−1^) (%; *n*)**	***Feed***	**(CFU·g^−1^) (%; *n*)**
*Cladosporium* sp.	71.4; 10	*Cladosporium* sp.	82.2; 37
*Penicillium* sp.	21.4; 3	*Penicillium* sp.	8.9; 4
*Fusarium culmorum*	7.1; 1	*Fusarium culmorum*	8.9; 4
**Floor covering**	**(CFU·g^−1^) (%; *n*)**	***Floor covering***	**(CFU·g^−1^) (%; *n*)**
*Penicillium* sp.	50; 4	*-*	-
*Alternaria* sp.	37.5; 3	*-*	-
*Cladosporium* sp.	12.5; 1	*-*	-

**Table 4:** *Aspergillus* sections’ distribution in air samples.

MEA		DG18	
Air	(CFU·m^−3^) (%; *n*)	Air	(CFU·m^−3^) (%; *n*)
*Circumdati*	55; 220	*Versicolores*	50; 240
*Aspergilli*	25; 100	*Usti*	20.8; 100
*Nigri*	10; 40	*Aspergilli*	12.5; 60
*Versicolores*	5; 20	*Candidi*	12.5; 60
*Flavi*	5; 20	*Nidulantes*	4.2; 20

**Table 5:** Results of the Kruskal–Wallis test for the comparison of fungi and bacteria concentration, both on surfaces and in air, between the five swine farms (*n* = 24).

Bacteria/Fungus	Swine Farming	*n*	Ranks	Test Statistics ^a^			Kruskal–Wallis Multiple Comparisons
			Mean Rank	Chi-Square	df	*p*	
Total Bacteria Surface (CFU·m^−2^)	A	5	12.00	1.936	4	0.748	
	B	5	10.00				
	C	5	14.10				
	D	4	10.75				
	E	5	15.30				
Gram Negative Bacteria-Surface (CFU·m^−2^)	A	5	12.40	0.081	4	0.999	
	B	5	12.60				
	C	5	12.00				
	D	4	12.75				
	E	5	12.80				
Fungi (MEA)-Surface (CFU·m^−2^)	A	5	17.90	13.699	4	0.008 *	C ≠ D (*p* = 0.036)
	B	5	12.50				
	C	5	6.00				
	D	4	19.50				
	E	5	8.00				
Fungi (DG18)-Surface (CFU·m^−2^)	A	5	18.60	8.430	4	0.077	
	B	5	13.60				
	C	5	10.30				
	D	4	8.50				
	E	5	10.70				
Total bacteria-Air (CFU·m^−3^)	A	5	12.10	3.676	4	0.452	
	B	5	14.10				
	C	5	10.40				
	D	4	17.50				
	E	5	9.40				
Gram Negative Bacteria-Air (CFU·m^−3^)	A	5	10.20	7.132	4	0.129	
	B	5	15.00				
	C	5	15.00				
	D	4	16.50				
	E	5	6.60				
Fungi (MEA)-Air (CFU·m^−3^)	A	5	6.40	17.602	4	0.001 *	A ≠ D (*p* = 0.007)
	B	5	8.20				B ≠ D (*p* = 0.025)
	C	5	9.40				
	D	4	22.50				
	E	5	18.00				
Fungi (DG18)-Air (CFU·m^−3^)	A	5	11.60	12.621	4	0.013 *	B ≠ D (*p* = 0.005)
	B	5	6.10				
	C	5	10.80				
	D	4	22.50				
	E	5	13.50				

^a^ Kruskal–Wallis test; * statistically-significant differences at the 5% significance level.
